# Gut microbiota and gut-derived metabolites in defining multiple sclerosis phenotypic continuum

**DOI:** 10.3389/fimmu.2026.1858047

**Published:** 2026-07-01

**Authors:** Federico Montini, Ashutosh Mangalam, Burcu Zeydan, Joseph Murray, Orhun H. Kantarci

**Affiliations:** 1 Department of Neurology, Mayo Clinic, Rochester, MN, United States; 2 Mayo Clinic Center for Multiple Sclerosis and Autoimmune Neurology, Rochester, MN, United States; 3 Department of Pathology, Carver College of Medicine, University of Iowa, Iowa City, IA, United States; 4 Iowa City Veterans Affairs Health Care System, Iowa City, IA, United States; 5 Division of Gastroenterology and Hepatology, Mayo Clinic, Rochester, MN, United States

**Keywords:** bile acids, gut-brain axis, metabolite, microbiota, multiple sclerosis, neuroinflammation, probiotic, short-chain fatty acids

## Abstract

Multiple sclerosis (MS) is a chronic inflammatory disease of the central nervous system in which environmental factors play an important role in shaping disease risk, activity, and progression. Over the past decade, human and experimental studies have consistently shown alterations in the gut microbiome across the phenotypic spectrum of MS and have linked these changes to immune dysregulation, barrier dysfunction, neuroinflammation, and demyelination. Additionally, emerging evidence indicates that microbial function, particularly metabolite production plays a more direct role in shaping immune responses and associated neuropathology. Evidence from both human studies and experimental autoimmune encephalomyelitis models supports a functional role for microbial metabolites in shaping neuroimmune responses. Bacterially derived metabolites such as short-chain fatty acids, bile acids, polyamines, phytoestrogen metabolites, and tryptophan-derived compounds can influence T-cell differentiation, glial activation, epithelial integrity, and neuroimmune communication. Recent longitudinal studies also show associations between metabolite profiles and disability worsening. Because disease-modifying therapies, diet, and microbiome-directed interventions can reshape microbial metabolism, microbial metabolites may represent promising therapeutic targets in the gut-immune-brain axis. In this *Review*, we integrate current evidence to propose a mechanistic framework in which microbial metabolites act as central regulators of mucosal and systemic immunity that influence different aspects of MS biology. We discuss how this perspective shifts gut microbiome research from descriptive associations to biological mechanisms that more directly link the gut to immune responses and downstream neuropathology. We then evaluate therapeutic strategies that target microbial metabolism and outline key priorities for longitudinal, multi-omics, and interventional studies that are needed to enable microbiome-informed precision therapies in MS.

## Introduction

**Figure 1:**
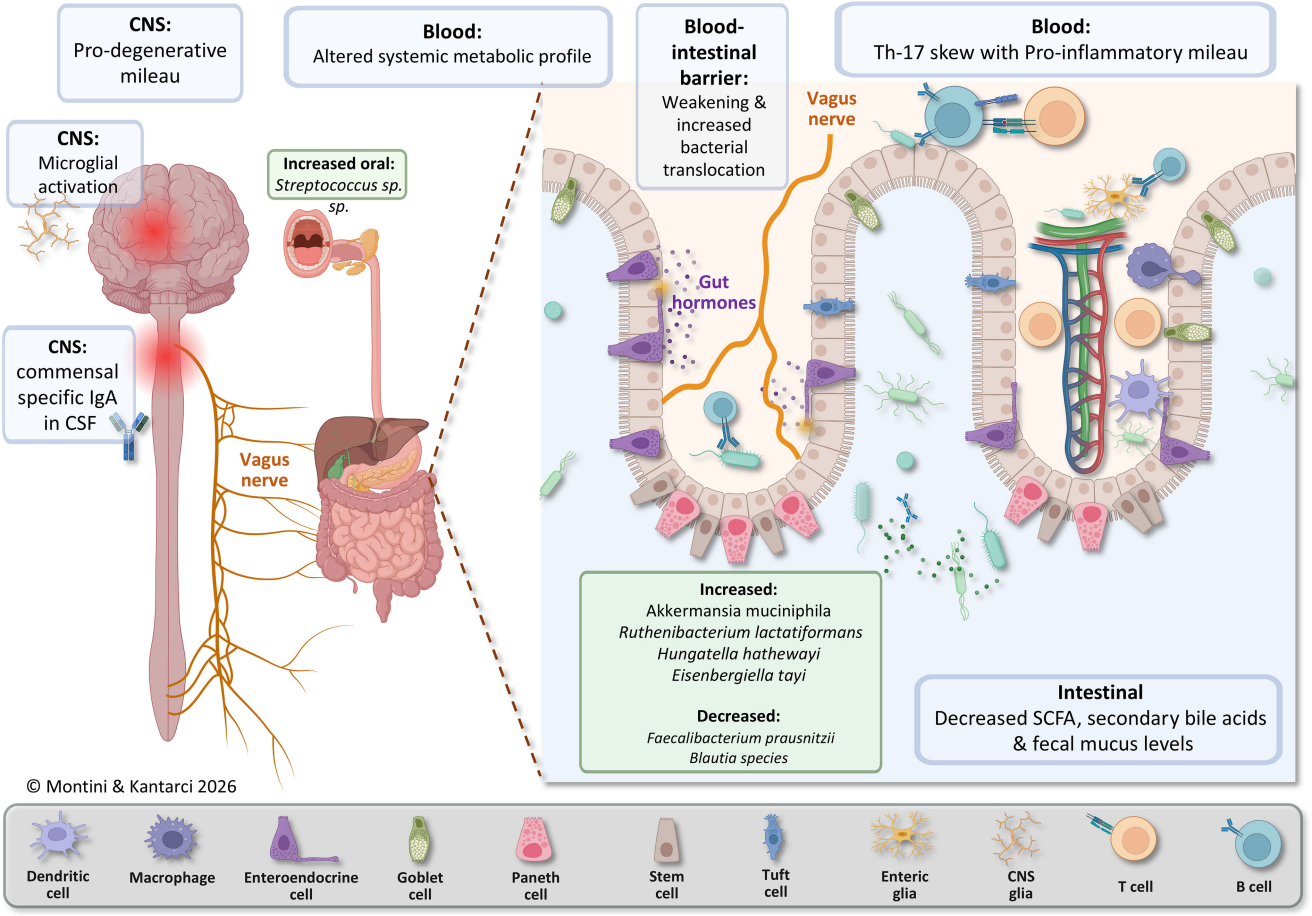
Schematic of healthy and multiple sclerosis intestinal environment. The image contrasts a healthy intestinal environment with alterations associated with multiple sclerosis (MS). Six interconnected domains of gut wall and gut–brain axis dysfunction are highlighted: (1) microbiome dysbiosis, including shifts in oral- and gut-derived taxa; (2) altered microbial and host metabolism, with reduced short-chain fatty acids, secondary bile acids, and fecal mucus levels, alongside changes in systemic metabolic profiles; (3) impaired epithelial tight junction integrity and intestinal barrier dysfunction, promoting increased bacterial translocation; (4) immune dysregulation, characterized by pro-inflammatory immune skewing, including Th17-associated responses; (5) enteric nervous system alterations; and (6) central nervous system immune activation, including microglial activation, commensal-specific IgA in cerebrospinal fluid, and a pro-degenerative CNS milieu. Together, these pathways illustrate potential mechanisms by which intestinal dysregulation may contribute to systemic inflammation and neuroinflammatory processes in MS. CNS, central nervous system; CSF, cerebrospinal fluid; IgA, immunoglobulin A; MS, multiple sclerosis; SCFA, short-chain fatty acids; Th17, T helper 17 cell.

Multiple sclerosis (MS) is a chronic inflammatory and demyelinating disease of the central nervous system in which environmental exposures play an important role in determining disease susceptibility, activity, and phenotypic evolution (1, 2). Among these environmental factors, the gut microbiome has emerged as a key modifiable regulator of immune homeostasis (3, 4). Over the past decade, human cohort studies and experimental models have consistently demonstrated alterations in gut microbial composition in MS and have linked these changes to immune dysregulation, barrier dysfunction, neuroinflammation, and demyelination (3, 5–10) (**Figure 1**).

Although microbiome alterations are consistently observed in MS, distinguishing causal microbial drivers from disease-associated changes remains a major challenge in the field (3, 5–10). As a result, much of the research to date has focused on identifying which microbes are present, whereas the biological mechanisms through which the microbiome influences neuroimmune disease have not been fully characterized. Increasing evidence now indicates that microbial function, particularly the production of bioactive metabolites, plays a more direct role in shaping mucosal and systemic immune responses and the associated neuropathology of MS (3, 4). These microbial signals can influence epithelial integrity, antigen presentation, T-cell differentiation, glial activation, and neuroimmune communication, thereby providing plausible mechanistic links between intestinal ecology and central nervous system inflammation (3, 4, 11–13).

MS is increasingly viewed as a phenotypic continuum rather than distinct relapsing or progressive diseases. Relapse-associated inflammation and progression-associated biology likely coexist in most patients, with the clinical phenotype reflecting which process predominates at a given time (14). This framework is relevant to microbiome research because gut-derived immune and metabolic signals may influence both relapse activity and disability progression.

Among the functional mediators, bacterially derived metabolites, including short-chain fatty acids, bile acids, polyamines, phytoestrogen metabolites, and tryptophan-derived compounds, have emerged as important regulators of systemic immune and innate neuroglial biology (3, 5). Longitudinal human studies further suggest that alterations in microbial metabolic profiles are associated with disability worsening and disease progression (5). Importantly, microbial metabolism is not static and can be reshaped by disease-modifying therapies, dietary exposures, and microbiome-directed interventions, highlighting the therapeutic relevance of targeting microbial metabolic pathways along the gut-immune-brain axis.

To develop microbiome-based treatments across various diseases, it is crucial to: i) define the host and disease-specific organization of microbial communities, ii) understand the microbial metabolism associated with the microbiome composition, and iii) characterize microbiome-host interactions. This review examines the growing body of evidence describing alterations in microbiome composition in MS and explores how these changes influence intestinal, systemic, and ultimately central nervous system immune responses. A deeper understanding of these mechanisms may facilitate the development of microbiome- and metabolite-based precision treatments in MS.

## The gut microbiota in multiple sclerosis

Alterations in the gut microbiota have been consistently observed in MS, with distinct microbial signatures emerging across the continuum of disease phases and phenotypes. Acute severe infections, such as COVID-19 which is associated with extensive pro-inflammatory cytokine surges (15–18), could impact the extent of acute inflammatory-demyelinating biology of MS in specific cases, however, they do not seem to have population-level impacts on the active relapsing disease phenotypes of MS. On the other hand, a more prominent smoldering effect of chronic colonization or infection associated with changes in the microbiome is more likely to have a lasting impact on generating population-level phenotypic variability in disease course (19). Alternatively, lower abundance organisms or their metabolites may impact the systemic immune responses by having direct effects via the small intestine which lacks the diverse and resilient community of the colon and the containment features of colon. The small intestine is both longer and has a much greater surface area where luminal host interface may be both a key portal for modification but also less dependent on changing the resilient microbial community of the colon. Focusing on the stool microbiome does not necessarily provide an accurate insight into the mucosal associated microbial community, especially those within the small intestine. **Table 1** summarizes the key differences in gut microbiota composition observed between people living with MS and healthy controls (HC), while **Table 2** summarizes the Disease-modifying-treatment (DMT) associated gut microbiome and metabolite changes.

**Table 1:** Summary of gut microbiome compositional differences across multiple sclerosis phenotypes.

MS phenotype and sample size	Lower abundance in MS	Increased abundance in MS	Geographical location and reference
RMS (n = 19) HC (n = 17)	*Prevotella* sp.	*Streptococcus* sp.	Italy (4)
RMS (n = 31) HC (n = 36)	*Prevotella* sp.*, Parabacteroides sp, Adlercreutzia* sp.*, Collinsella* sp.*, Lactobacillus*	*Pedobacter* sp.*, Pseudomonas* sp.*, Mycoplasma* sp.*, Haemophilus* sp.*, Blautia* sp.*, Dorea* sp.	USA (6)
RMS (n = 60) HC (n = 43)	*Butyricimonas* sp.*, Prevotella* sp.*, Parabacteroides* sp.	*Methanobrevibacter* sp.*, Akkermansia* sp.	USA (7)
RMS (n = 30) HC (n = 14)	*Eubacterium eligens, Prevotella copri*, uncultured Bacteroides sp., uncultured alpha Proteobacterium, uncultured Pseudomonas sp.	*Faecalibacterium* sp.*, Ruminococcus* sp., uncultured Oscillospiraceae sp., uncultured Blautia sp., *Anaerostipes* sp.*, Clostridium bolteae*, uncultured Dialister sp., *Alistipes onderdonkii, Bifidobacterium longum, Coriobacterium* sp.	UK (160)
RMS (n = 71) HC (n = 71)	*Parabacteroides distasonis*	*Akkermansia muciniphila, Acinetobacter calcoaceticus*	USA (35)
RMS (n = 24) HC (n = 25)	*Bifidobacterium longum, Clostridium leptum, Faecalibacterium prausnitzii, Bacteroides thetaiotaomicron*, unclassified Escherichia, *Anaerostipes* sp.*, Prevotella* sp.	*unclassified Parabacteroides*	USA (161)
RMS (n = 20), HC (n = 40)	*Bacteroides (B. stercoris*,B. coprocola, B. coprophilus), *Fecalibacterium* sp.*, Prevotella (P. copri), Anaerostipes* sp.*, Clostridium* sp.*, Sutterella (S. wadsworthensis)*	*Bifidobacterium* sp.*, Streptococcus* sp.*, Streptococcus thermophilus, Eggerthella lenta*	Japan (8)
*Monozygotic twin pairs:* MS* (n = 34), Unaffected twin (n = 34)	*Adlercreutzia* sp.	*Akkermansia* sp.	Germany (162)
Pediatric onset MS (n = 20), HC (n = 20)	*Pseudomonas corrgata, Haemophilus influenzae, Kochuria* sp.*, Sphingopyxis* sp. *QXT-31*	*Methanobrevibacter smithii, Moribacter* sp.*, Mycoplasma bovoculi, Diaphorobacter Polyhydrobutyrativorans, Methanobrevibacter millerae, Arcanobacterium* sp. *2701*	Canada (163)
RMS (n = 20), HC (n = 30)	*Barnesiella* sp.*, Odoribacter* sp.*, Oscillospiraceae UCG-003*	*Blautia* sp.*, Eggerthella* sp.*, Hungatella* sp.	USA (164)
MS (n = 576), Paired HC (n = 1152)	*Faecalibacterium prausnitzii, Blautia species*	*Akkermansia muciniphila, Ruthenibacterium lactatiformans, Hungatella hathewayi, Eisenbergiella tayi*	USA, Spain, UK, Argentina (30).
RMS = 199, HC = 40	*Blautia wexlerae; Dorea formicigenerans; Erysipelotrichaceae CCMM*	*Akkermansia muciniphila; Clostridium bolteae; Ruthenibacterium lactatiformans*	USA (28)
PMS = 44, HC = 40	*Blautia wexlerae; Dorea formicigenerans; Blautia (unclassified); Agathobaculum*	*Akkermansia muciniphila; Enterobacteriaceae; Clostridium g24 FCEY; Streptococcus; Clostridium bolteae*	USA (28)
Monozygotic twin pairs: MS (n = 81); Unaffected co−twin (n = 81)	*Dialister succinatiphilus; Prevotella buccae*	*Eisenbergiella tayi; Copromonas; Acutalibacter; Anaerotruncus massiliensis; Alistipes ihumii*	Germany (33)
RMS (n = 45); HC (n = 51)	*Prevotella copri, Faecalibacterium prausnitzii, Bifidobacterium adolescentis*	*Blautia* spp. *(e.g., Blautia* sp. *CAG:257), Akkermansia* spp.*, Collinsella, Eggerthella, Dorea* sp. *CAG:317, multiple Ruminococcus* spp. *(e.g., [Ruminococcus] gnavus, [Ruminococcus] torques)*	USA (32)

**Table 1** summarizes published human studies comparing gut microbiota composition in individuals with multiple sclerosis (MS) and healthy controls (HC). For each cohort, the table lists the MS phenotype studied, including relapsing MS (RMS), progressive MS (PMS), pediatric-onset MS, and MS* when the symptomatic phenotype was not specified—alongside sample size, bacterial taxa reported at lower or higher abundance in MS relative to HC, and the corresponding geographical location and reference. Taxonomic shifts reflect study-specific sequencing platforms and analytic methods. Across cohorts, recurrent findings include reduced abundance of short-chain-fatty-acid–producing taxa (e.g., *Faecalibacterium*, *Blautia*, *Prevotella*) and increased abundance of mucin-degrading or pro-inflammatory taxa (e.g., *Akkermansia*, *Methanobrevibacter*, *Clostridium bolteae*), though results vary by region, methodology.

MS, multiple sclerosis; RMS, relapsing MS; PMS, progressive MS; POMS, pediatric-onset MS; MS*, MS phenotype not specified; HC, healthy control; SCFA, short-chain fatty acid.

**Table 2:** Disease-modifying-treatment-associated gut microbiome and metabolite changes.

DMT	Core human evidence (study/design, n)	Taxa-level changes (direction)	Metabolite/functional signals	Diversity changes
Dimethyl fumarate (DMF; oral)	Cross-sectional RMS; DMF vs GA vs naïve (Katz-Sand n=168) (47). Multicentric cohort Zuo, n=576 (30). Observational cohort (Stuan-Ram, n=45) (165)	↓ *Lachnospiraceae, Veillonellaceae Firmicutes/Fusobacteria; Clostridiales; ↑ Roseburia*	Predicted pathway shifts (PICRUSt). Independent cohorts: ↑ TCA-cycle metabolites on DMF; baseline gut composition associates with DMF-induced lymphopenia risk (Diebold) (55). iMSMS shows treatment-linked functional changes across pathways.	No consistent α-change; modest β-shifts by therapy (study-specific). DMT use vs non-use generally shows no α-diversity difference (iMSMS).
Teriflunomide (oral; DHODH inhibitor)	2-mo longitudinal (IFN-β or teriflunomide); n=39 (166);Multicentric cohort Zuo, n=576 (30).	Post-treatment ↑ Lachnospiraceae, ↑ *Streptococcus*; over time ↑ *Bifidobacterium angulatum*, ↓ *Oscillospira*; baseline MS vs HC: ↑ *Prevotella stercorea*, ↓ *Faecalibacterium prausnitzii*.	–	No significant α/β changes.
Cladribine (oral; immune reconstitution)	Multicenter pilot (BIA): baseline/3 mo/12 mo; n=25 RMS (48).	Limited global changes; oral ↓ Bacteroidetes at 12 mo; responders: ↑ *Faecalibacterium prausnitzii*, ↑ *Prevotella*, ↓ *Proteobacteria* vs non-responders.	–	No significant α/β changes
S1P modulators (fingolimod,oral)	Multicentric cohort Zuo (30), n=576. + systematic review, Tsai (167).	↑ *Clostridium* sp. *CAG:352 and Ruthenibacterium lactatiformans*, ↓ *Roseburia*, and *Blautia*	–	No significant α/β changes
Glatiramer acetate (GA; SC)	Cross-sectional RMS; DMF vs GA vs naïve (Katz-Sand, n=168) (47). Multicentric cohort Zuo, n=576 (30).	*↓ Lachnospiraceae; ↓ Veillonellaceae*	Overlapping predicted functional changes with DMF (PICRUSt).	No significant α/β changes
Interferon-β (IFN-β; SC/IM)	Multicentric cohort Zuo (30), n=576. + systematic review, Tsai (167).	↑ *Akkermansia* and *Parabacteroides distasonis* *↓ Ruminococcus, Clostridium, Faecalibacterium*, and *Roseburia.*	iMSMS: upregulation of SCFA transporter pathways in IFN-β users; consistent with reports of altered SCFA handling under therapy.	No significant α/β changes
Anti-CD20 (e.g., ocrelizumab; IV)	Longitudinal MS cohort Troci (49), n=36 Multicentric cohort Zuo, n=576 (30).	↓ pro-inflammatory Gram-negative bacteria (e.g., *Escherichia*/*Shigella*). ↑ *Faecalibacterium*.	–	↑ α-diversity in gut & oral after B-cell depletion, persisting longitudinally, Troci, no change in iMSMS
Natalizumab (IV; anti-α4 integrin)	Multicentric cohort Zuo, n=576 (30).	↑*Prevotella* and *Bacteroides* coprophilus↓ Ruminococcus, Collinsella	–	No significant α/β changes

**Table 2** compiles human studies reporting how multiple sclerosis disease-modifying therapies (DMTs) are associated with gut microbiome taxa-level and functional/metabolite changes, alongside diversity metrics. Microbial taxa are *italicized*. Arrows denote direction of change relative to each study’s comparator (↑ increase, ↓ decrease). Diversity terminology follows standard usage: α-diversity (within-sample richness), β-diversity (between-sample dissimilarity). Study designs, comparators, and sequencing modalities (16S rRNA vs shotgun) are reported as in the original publications.

DMF, dimethyl fumarate; GA, glatiramer acetate; DMT, disease-modifying therapy; HC, healthy control; IFN-β, interferon-beta; iMSMS, International Multiple Sclerosis Microbiome Study; RMS, relapsing multiple sclerosis; SCFA, short-chain fatty acid; S1P, sphingosine-1-phosphate; NTZ, natalizumab; TCA cycle, tricarboxylic acid cycle.

### Relapsing MS

Early evidence (6) from a North American cohort of 31 patients with symptomatic relapsing MS (RMS) and 36 HC showed that patients with RMS harbor a distinct gut microbiota in their feces compared to HC, characterized by increased abundance of *Blautia, Dorea, Pseudomonas, and Mycoplana*, and a reduced abundance of *Parabacteroides, Adlercreutzia, and Prevotella*. The study together with more recent work (N: 20 RMS, 20 HC) (3) highlighted a depletion of bacteria involved in secondary bile acids metabolism (BAM), and Deoxycholic acid (DCA) and lithocholic acid (LCA) play key immune regulatory functions by promoting T regulatory cell differentiation at the expense of Th17 cells (3). Other studies have reported increased abundance of *Akkermansia* and decreased levels of butyrate-producing taxa such as *Blautia, Faecalibacterium*, and *Oscillospiraceae* (6–10). Butyrate is a short-chain fatty acid (SCFA), and decreased levels of butyrate can be associated with Alzheimer’s disease (20), ulcerative colitis, and with fatigue (21). These compositional shifts are associated with increased intestinal Th17 cell frequency and increased disease activity in MS (4). Additionally oral bacteria, such as Streptococcus (22), are overly expressed in MS. Interestingly oral bacteria are known to exacerbate intestinal inflammation (23). The link between microbial dysbiosis and the MS immunobiology, such as the Th17 skew associated with disease activity even in patients on high-efficacy disease modifying therapy (24) would implicate a relatively direct pathway from microbiota to MS phenotypic continuum related to the relapses.

### Progressive MS

Recent efforts have extended microbiome profiling to the symptomatic progressive phase of MS (PMS), revealing a different microbiota signature from HCs and RMS with some potential regional variability. An eastern European cohort of patients presenting with PMS [N: 15 PMS, 15 HC] showed increased *Akkermansiaceae* (25), while a Western European (N: 26 PPMS, 120 HC) and an East Asian [N: 15 PMS, 55 HC] cohort reported reduced butyrate producers and increased *Methanobrevibacter* and *Clostridium* species (26, 27). A North American cohort (N: 44 PMS, 40 HC) identified elevated Enterobacteriaceae and Clostridium bolteae (28), both known to promote Th17 cell differentiation (29), alongside increased *Akkermansia*, which conversely was associated with a protective effect in an animal model of MS (28). The International Multiple Sclerosis Microbiome Study (iMSMS), a worldwide multi-center cohort of 576 people living with MS and matched household controls, confirmed several of these findings, including increased *Akkermansia* and *Ruthenibacterium* and decreased *Blautia* and other butyrate producers (30). In a newer North American study (N: 124 stable RMS, 68 who transitioned to symptomatic PMS) (5) we demonstrated that individuals transitioning from symptomatic RMS to symptomatic PMS over a two-year period exhibited loss of SCFA-producing microbes such as *Eubacterium hallii, Butyricicoccaceae*, and *Blautia*. *Alistipes* were associated with worsening disability, higher rate of whole brain atrophy on MRI, and cognitive decline.

In a longitudinal North American cohort (31) of 58 individuals with MS followed for 4.2 ± 1.0 years, baseline stool 16S profiles were compared between patients who later progressed (n = 14; ΔEDSS > 0.5) and non-progressors (n = 44). Although global alpha/beta diversity differences were modest, progressors showed depletion of *Akkermansia muciniphila* and multiple SCFA-producing taxa in *Lachnospiraceae/Oscillospiraceae*, and enrichment of *Alloprevotella*, *Prevotella-9, Bilophila, Sutterella*, and *Alphaproteobacterial Rhodospirillales*. Inferred metagenomics indicated increased ubiquinone/aerobic-respiration pathways with reduced menaquinone (vitamin K_2_) and SCFA biosynthetic capacity, consistent with a more oxidative luminal environment.

In a recent North American study (32), we identified a reduced ratio of *Bifidobacterium adolescentis* to *Akkermansia muciniphila* (BA: AM) as a microbial marker of disease severity in MS. In this cohort, MS was characterized by evidence of both intestinal and systemic inflammation, including elevated fecal lipocalin-2 (LCN2) and plasma osteopontin levels. Notably, a lower BA: AM ratio correlated with higher disability on the Expanded Disability Status Scale (EDSS)(27) and with increased inflammatory markers, linking microbial imbalance to both clinical severity and inflammatory activity.

In experimental models, administration of the MS-associated strain *Blautia wexlerae* reduced the BA: AM ratio, increased gut inflammation consistent with findings in people with MS, worsened Experimental Autoimmune Encephalomyelitis (EAE) severity, and enhanced IL-6 and IL-12/23 production (32). These findings support a functional relationship between microbial imbalance and immune activation.

In the iMSMS cohort (30), the BA: AM ratio was similarly lower in MS compared with household controls and was inversely associated with EDSS scores and disease duration, independently validating its association with disease severity across populations.

A recent monozygotic-twin study (33) identified 51 differentially abundant taxa, with MS-affected twins showing reduced propionate-producing species such as *Dialister succinatiphilus* and *Prevotella buccae*, and increased *Eisenbergiella tayi* and other *Firmicutes* in the ileum, which were not detectable in stool. Functional transfer experiments demonstrated that ileal microbiota from MS-affected twins, but not their healthy co-twins, induced spontaneous EAE in germ-free mice, driven by expansion of *Lachnospiraceae* members (*Eisenbergiella tayi* or *Lachnoclostridium*) (33). These findings highlight disease-relevant, small-intestinal taxa capable of triggering Th17-skewed CNS autoimmunity.

A dominant theme across all studies is the finding of increased *Akkermansia* in MS regardless of the symptomatic phase the patient is in. *Akkermansia* can directly modulate microglia (34) and has been reported as negatively associated with disability accumulation in people living with MS and with EAE severity in mice (28), although other studies suggested an opposite effect (32, 35). Despite these conflicting results, they are intriguing since in MS, intestinal mucus is reduced, and mucus levels are associated with mucin degrading bacteria, such as *Akkermansia* (13).

These findings suggest that microbial balance, rather than the presence of individual taxa, may be critical in MS pathogenesis.

## Sex-specific differences in the microbiota and its metabolites in multiple sclerosis

Biological sex can influence disease risk, course, and severity, in MS (36). The female-to-male ratio is around 3:1 and men typically develop symptoms at a later age but are more prone to earlier symptomatic progression (37–39). Interestingly, androgen modifying therapy may increase the risk of disease activity in older men (40), earlier menopause is linked to increased symptomatic progression manifestation (41), and menopause and male sex are associated with higher levels of upper spinal cord atrophy (42), suggesting a direct role of sex hormonal-immune system interactions driving the phenotypic MS continuum in part (38, 43).

Pubertal studies have shown that sex-specific differences in microbiome arise during puberty and persist into adulthood (44). In MS, sex was reported as the second strongest variable associated with differences in oral and nasal microbiota (22). Moreover, bacterially-derived metabolites including SCFAs vary by sex (45). These observational studies suggest that sex differences are likely to contribute to the variability in how the microbiome and its metabolites impact MS disease continuum, highlighting the need for further investigation specifically addressing sex as a biological variable in MS microbiome.

## Interaction of DMTs with gut microbiota and metabolism

Disease-modifying therapies (DMTs) are essential in MS management, reducing relapse rates and slowing disability worsening through decreasing relapse associated worsening (RAW). Emerging evidence indicates that DMTs also influence gut microbiota composition and function, potentially contributing to their therapeutic effects (30, 46). Key DMT-associated changes in gut microbiota composition are reported in **Table 2**.

### The route of administration impacts DMT effect on microbiota

Oral agents like dimethyl fumarate (DMF) and fingolimod decrease *Bacteroides*, *Blautia*, and *Clostridium* species, whereas parenteral drugs such as glatiramer acetate, interferon-β (IFN-β), anti-CD20 therapies, and natalizumab reduce *Faecalibacterium prausnitzii, Dialister invisu*s, and *Roseburia intestinalis* (30). While the global microbiota composition and the beta diversity remains largely unchanged (28, 30), genus-level shifts are common.

DMF, a potent microbiota modulator, lowers *Bacteroides stercoris, Clostridia, Eubacterium, Lachnospiraceae, Veillonellaceae, and Tissierellaceae*, while increasing *Roseburia intestinalis* (28, 30, 47). Fingolimod reduces *Bacteroides finegoldii*, *Roseburia faecis*, and *Blautia*, but raises *Ruminococcaceae* (28, 30).

Natalizumab treatment increased *Phascolarctobacterium* sp. CAG:207, while decreasing several Prevotella species and *Bifidobacterium longum* (30), exacerbating their depletion known in MS. On the other hand, one year after cladribine treatment (48), *Bacteroidetes, Prevotella, and Faecalibacterium prausnitzii*, which are known to be depleted in untreated MS (30), are restored.

IFN-β decreases *Ruminococcus, Clostridium, Faecalibacterium*, and *Roseburia*, while increasing *Parabacteroides distasonis* (30). Natalizumab elevates *Phascolarctobacterium*, *Ruminococcaceae* and *Prevotella*, but reduces *Bifidobacterium longum* (28, 30). Anti-CD20 therapy decreases *Bacteroides, Blautia, Escherichia, Parabacteroides, Bifidobacterium Adolescentis, and Prevotella*, while increasing *Faecalibacterium* (28, 30, 49).

Together, these data suggest that DMT-associated microbiome changes differ by route of administration, with oral agents primarily linked to shifts in *Bacteroides*, *Blautia*, and *Clostridium*, and parenteral therapies associated with changes in SCFA-producing or immunoregulatory genera such as *Faecalibacterium*, *Roseburia*, *Dialister*, *Prevotella*, and *Bifidobacterium*, despite largely preserved overall microbiota structure.

## DMTs affect gut-derived metabolites through host–microbe interactions

IFN-β treatment reduces metabolites linked to amino acid, carbohydrate, nucleotide, and energy metabolism (30). DMF shows similar effects (47). Glatiramer acetate has minimal metabolic impact (30, 50). Fingolimod lowers pyruvate in feces and serum (30). SCFAs, particularly acetate and propionate, are consistently reduced in people living with MS regardless of DMT (30).

Propionate supplementation improves Treg/Th17 balance and clinical outcomes (51), and IFN-β may enhance propionate absorption, explaining observed serum increases (30).

Overall, these findings indicate that DMT-associated metabolite changes are incompletely characterized, and current evidence remains insufficient to determine whether observed metabolic differences reflect direct treatment effects, secondary immune modulation, or the persistent MS-associated depletion of key microbial metabolites.

## Microbiota changes may influence DMT efficacy

Some agents, including teriflunomide, DMF, and fingolimod, exhibit antimicrobial activity (52, 53), while fumarate may serve as a bacterial substrate and affect antibiotic resistance (54). DMF-induced lymphopenia and gastrointestinal side effects have been linked to microbiota alterations (55, 56).

Moreover, gut microbes can modulate responses to immunotherapies, as shown in cancer patients where *Akkermansia muciniphila* abundance restored checkpoint inhibitor efficacy (57). Similar mechanisms may apply to MS, warranting further investigation into microbiota-driven variability in DMT response.

## The intestinal immune system in MS

The intestinal wall provides a physical and biochemical barrier that limits microbial access to host tissues while maintaining immune homeostasis and oral tolerance via the controlled interaction with the commensal organisms. Although some studies report increased intestinal permeability in subsets of people living with MS, evidence remains inconsistent, and widespread epithelial disruption is not uniformly observed (11, 12). Recent work suggests that the epithelial tight-junction barrier is largely intact, whereas the mucus layer, primarily composed of MUC2, is significantly reduced in both RMS and PMS, correlating with enrichment of mucin-degrading taxa such as *Akkermansia* and *Bacteroides* (13). Another work (58) recently described an alteration of the enteric nervous system and specifically enteric gliosis in PMS. This suggests a global alteration of the intestinal environment in MS, with weakening of the mucus which may lead to a tighter microbiota-immune system interaction (59) and potentially to a Th17 skew (4). Interestingly, natalizumab, one of the highest-efficacy DMTs for MS, inhibits α4 integrin–mediated leukocyte trafficking, highlighting the importance of immune cell migration pathways in CNS autoimmunity (60). Consistent with a gut-immune axis contribution, natalizumab is also effective in Crohn’s disease and ulcerative colitis, conditions characterized by disruption of intestinal barrier integrity, as well as in celiac disease (60, 61).

The gastrointestinal tract, beyond serving as a physical barrier, is the body’s largest immune organ and plays a central role in shaping systemic immunity. In MS, environmental factors (62) and a perturbation of the gut microbiota and of the gut-blood barrier influence immune function (63), including effects on gut-derived Mucosal-Associated Invariant T (MAIT) cells (64, 65) and Th17 cells (4). In MS, alterations in gut microbiota composition is associated with increased frequency of Th17 cells in the small intestine, which correlate with longitudinal disease activity (4). Enteric glia, the most common glial cell in the intestinal nervous system, can function as antigen presenting cells, and their hyperplasia has been observed in PMS (58).

In EAE, gut-resident Th17 cells respond to microbial stimuli and can become pathogenic, migrating to the central nervous system (CNS) (66–69). These myelin-reactive Th17 cells accumulate in the colon through α4β7–MAdCAM-1–dependent trafficking pathways (70), and their disease-driving potential is influenced by microbial metabolites, as long-chain fatty acids promote Th17 responses whereas short-chain fatty acids support regulatory T cell differentiation (67). IL-17 isoforms are critical in disease severity: deleting IL-17A/F reduces EAE symptoms, while reintroducing IL-17A at the gut mucosa restores pathology (71). Dietary fiber and microbiota-derived SCFAs influence the Th17/Treg balance, highlighting diet–microbe–immune interactions as therapeutic targets (72).

Gut wall immune homeostasis during EAE has been less extensively studied beyond T cells, however, some studies have been undertaken to discern the role of B-cells, and specialized intraepithelial T cells (IELs). Importantly, substantial cellular or cytokine inflammation is not observed in the intestinal wall at the onset or during EAE (73). In the 2D2 transgenic mouse model, mice who developed EAE showed higher number of IELs compared with animals that did not develop symptomatic EAE (73). Furthermore, 2D2-TCR high expressing cells produced more IL-17A, IFNγ, and IL-10 compared to splenic T cells and IELs expressing low levels of the 2D2-TCR (73). In parallel, patients with PMS showed reduced frequencies of CCR9+ CD4+ T cells, a cell population with gut-homing and IEL potential, and increased production of IL-17 and IFN-γ compared to healthy controls (74).

B cells are another critical component of the gut immune system with antigen presentation to autoreactive T cells being the commonly proposed mechanism in MS immunopathogenesis (1, 2). The role of gut-resident B cells in MS, however, remains less well defined. B cells producing microbiota-specific IgA have been shown to traffic to the CNS in people living with MS undergoing an active relapse (75). Similarly, IgA+ plasma cells (PCs) have been shown to traffic from the gut to the CNS during EAE (76). Interestingly in this study, CNS PCs carrying gut-derived IgA appeared to exert beneficial effects, as their depletion exacerbated EAE, whereas restoration of IgA responses in the gut mucosa ameliorated disease severity (76). These findings underscore the importance of mucosal IgA responses in modulating neuroinflammation.

Recent mechanistic studies extend this concept to glial regulation: mono-colonization of germ-free mice with *Akkermansia muciniphila* strains altered microglial and astrocyte gene expression, including pathways related to immune signaling and myelination, and skewed peripheral T-cell phenotypes toward a less inflammatory profile (34). These effects were strain-specific and associated with SCFA production, particularly propionate, which has been linked to beneficial immune modulation in MS (34).

Further elucidation of mechanisms by which microbiota and their products interact with the gut mucosal immune system is needed to define relationships driving both homeostasis and pathology.

### Gut microbial metabolites as potential therapeutics in MS

Microbes carry out numerous functions critical to host health through the production and secretion of metabolites (77). Nutrient breakdown by gut microbes generates metabolites from dietary macronutrients that are transported or absorbed into the gut wall (78). Studies using germ-free mice showed drastic changes in serum amino acid metabolites compared to wild type mice (79). Germ-free mice and rats have significantly reduced levels of serum SCFAs (80), and germ-free mice have a dysregulated bile acid homeostasis leading to lower secondary bile acids levels (81). Research using germ-free mice underscores the essential role of microbial metabolites in maintaining host homeostasis. Gaining insight into the composition and functional impact of these metabolites, particularly in diseases such as MS, is vital for developing targeted microbial and metabolic therapies.

### Short-chain fatty acids and Indoles

SCFAs are among the best-characterized gut microbial metabolites, with well-documented physiological and immunological roles (72). They function as signaling molecules, energy sources, and modulators of epigenetic processes (82), influencing host metabolism, immunity, and the nervous system. In MS, serum SCFA levels including butyrate (83), propionate, and acetate are reduced compared to healthy controls (84–86). Lower SCFA concentrations have also been linked to disability worsening in both RMS and PMS (27, 87).

Evidence suggests SCFAs modulate immunity, as propionate supplementation in people living with MS increased Tregs and IL-10 while reducing Th17 cells in the gut and CNS, correlating with slower disability worsening over three years (51). Notably, while fecal concentrations of acetate, propionate, butyrate, isobutyrate, isovalerate, and valerate were not associated with 2-year EDSS worsening or DMT status, SCFA-producing taxa (e.g., *Eubacterium hallii*, *Butyricicoccus*) were associated with 2-year EDSS worsening and *E. hallii* correlated with favorable Neuro-QoL domains (5). Moreover, butyrate producers such as *Roseburia* had a negative correlation with leptomeningeal enhancement foci and showed a protection from EAE (59). However, the precise mechanisms by which SCFAs modulate mucosal immunity and confer benefit to people living with MS remain to be fully defined.

In EAE, SCFAs have shown therapeutic potential. Oral butyrate before disease onset reduced severity, while post-onset treatment had modest effects (88). SCFAs appear to promote Treg differentiation, whereas medium- and long-chain fatty acids favor Th1 and Th17 responses (67). These effects likely involve epigenetic and metabolic regulation of immune cells. Future research should explore SCFAs as therapeutic agents through longitudinal, controlled trials accounting for disease-modifying therapies and antibiotic use.

Indoles are microbiota-derived tryptophan metabolites that may represent an additional mechanistic link between gut dysbiosis, mucosal immunity, and CNS inflammation in MS. Several indole derivatives, including indole-3-aldehyde, indole-3-acetic acid, and indole-3-propionic acid, act through host receptors such as the aryl hydrocarbon receptor (AhR) and pregnane X receptor (PXR), promoting epithelial barrier integrity, IL-22–mediated mucosal protection, and anti-inflammatory immune signaling (89, 90). In experimental models, microbial tryptophan metabolites suppress CNS inflammation by activating AhR signaling in astrocytes (89), while indole-3-propionic acid regulates intestinal barrier function through PXR and TLR4-dependent pathways (91). The translational relevance of this pathway is further supported by an ongoing randomized clinical trial testing oral indole-3-propionic acid supplementation in RMS (clinicaltrials.gov; NCT07318129).

### Bile acids

Bile acids (BAs) emulsify dietary lipids to enable intestinal absorption and are thus fundamental to gastrointestinal and systemic physiology, yet they also act as host-microbiome–modulated signaling molecules. Antibiotic exposure perturbs the diversity of the BA pool in mice, underscoring microbial control over BA composition (92). Among microbially shaped species, isoallolithocholic acid inversely associates with inflammatory bowel disease severity (93), via inhibition of Th17 differentiation (94). BA signaling additionally calibrates innate immunity: receptors such as Farnesoid X receptor (FXR) on myeloid cells restrain TLR pathways, thereby limiting monocyte/macrophage inflammatory activation (95, 96). Because microbial transformations generate secondary bile acids (BAMs) that both support fat handling and tune immune phenotypes, they are attractive candidates for gut-targeted interventions.

In neuroinflammation models, metabolomics demonstrate that taurine-conjugated BAs rise during EAE progression in parallel with lipid remodeling, oxidative stress, and heightened inflammation (97); these shifts likely reflect metabolic activities of taxa such as Bacteroidetes and Bilophila wadsworthia, which process taurine-conjugated BAs (98, 99).

In people with MS, BA metabolism is altered: A) circulating BA levels are lower than in healthy controls, B) BA receptors are upregulated on lesion-resident macrophages/myeloid cells and astrocytes, and C) tauroursodeoxycholic acid (TUDCA) attenuates neurotoxic A1 astrocyte and M1-like microglial states *in vitro* (100). Consistently, pharmacologic FXR activation with obeticholic acid in EAE diminishes disease severity, inflammatory cytokines, and lymphocyte activation, reduces CD4^+^, CD8^+^, and B-cell populations, and renders Th17 cells incapable of transferring disease upon adoptive transfer (101). Extending these foundations, a shotgun-metagenomics and metabolomics study (3) showed that RMS is marked by a deficit of bacteria carrying the 7α-dehydroxylation machinery with a corresponding drop in intestinal deoxycholic acid (DCA), alongside an expansion of peripheral Th17 cells; RMS fecal filtrates drove Th17 differentiation *in vitro*, which exogenous DCA reversed, and oral DCA/lithocholic acid (LCA) prophylaxis in EAE curtailed clinical disease, reduced demyelination and axonal loss, expanded FOXP3^+^ Tregs and Tr1 cells, and suppressed encephalitogenic Th17/Th1 (including GM-CSF^+^).

In symptomatic progressive MS, a randomized, placebo-controlled TUDCA trial demonstrated safety and biological activity: supplementation (2 g/day, 16 weeks) shifted circulating BA profiles, reduced central memory CD4^+^ and Th1/Th17 cells, increased naïve CD4^+^ cells, and reprogrammed gut microbial metabolism with upregulated NAD salvage and downregulated mucolytic and purine-degradation pathways, consistent with a move toward less inflammatory immunity and altered microbiome function (102). Complementing these results, a two-year longitudinal cohort linked MS worsening and transition to symptomatic progression with lower serum ursodeoxycholate and isoursodeoxycholate, higher p-cresol sulfate and palmitoleate, and broad reductions in stool metabolites notably nicotinate (vitamin B3) suggesting a progressive loss of potentially protective microbial metabolites and a divergence between gut and blood compartments (5).

Collectively, the evidence indicates that microbe-derived secondary BAs actively shape innate and adaptive immunity in MS and EAE, are perturbed as disease advances, and can be therapeutically manipulated, via strategies that restore 7α-dehydroxylation capacity, dietary approaches, or direct BA supplementation (e.g., TUDCA), while future work should prioritize longitudinal sampling across the full phenotypic disease continuum of MS with integrated metagenomics, metabolomics, and immune readouts to pinpoint causal BA pathways and enable microbiome-based precision therapies.

### Phytoestrogens

Phytoestrogens are plant-derived polyphenols, principally isoflavones from soy and lignans from flaxseed, that resemble human estrogens; although humans cannot metabolize them directly, specific gut microbes convert isoflavones such as daidzein and genistein into bioactive metabolites (notably S-equol and O-DMA), with genera including *Adlercreutzia, Bifidobacterium, Eggerthella, Lactobacillus, Slackia, Prevotella*, and *Parabacteroides* implicated, several of which are reduced in people living with MS (103, 104). Phytoestrogens and their metabolites engage estrogen receptors ERα and ERβ on human and murine immune cells (T cells, B cells, NK cells, macrophages, and dendritic cells), and S-equol exhibits strong estrogenic, antioxidant, and anti-androgenic activities, collectively supporting local and systemic immunomodulation (104, 105). Unsurprisingly, given their estrogen like effects, these have been used to reduce menopausal symptoms (103, 104). Isoflavones also exert gut anti-inflammatory effects and protect against EAE in a microbiota-dependent manner, requiring isoflavone-metabolizing bacteria capable of producing S-equol; isoflavone diets enrich beneficial taxa, modulate lipopolysaccharide (LPS) biosynthesis pathways, and alter phenylalanine and lipid metabolism to lessen disease severity (104, 106, 107). Overall, the observed depletion of phytoestrogen-metabolizing bacteria in MS suggests impaired generation of beneficial metabolites like S-equol, and highlights diet–microbiome–metabolite interactions as promising therapeutic targets, though studies directly linking phytoestrogen levels or supplementation to MS disease course, particularly during perimenopausal transition, remain limited and are needed (104, 106, 107).

### Polyamines

Polyamine metabolites exert diverse effects on gastrointestinal health and immunity and have been implicated in multiple physiological processes as well as diseases such as cancer and Alzheimer’s disease (108, 109). In the gut, polyamines can be synthesized by host cells or by several microbial taxa, including *Bacteroides thetaiotaomicron, Fusobacterium varium, Enterococcus*, and *Bifidobacterium* spp (84, 110, 111). Functionally, polyamines contribute to maintenance of the intestinal barrier: they induce TLR2 expression on intestinal epithelial cells, and pharmacologic blockade of polyamine pathway flux increases epithelial permeability *in vitro* (112, 113).

Polyamines are also central regulators of immune cell trajectory. In CD4^+^ T cells, polyamines help direct lineage decisions in MS relevant pathways (62) by inducing c-Myc via Akt–mTOR signaling; c-Myc, in turn, drives expression of ornithine decarboxylase and other enzymes in polyamine biosynthesis (114, 115). Consistent with this, genetic knockouts of polyamine enzymes dysregulate T-cell differentiation (116), underscoring a mechanistic role for this pathway in immune homeostasis. Several lines of evidence implicate polyamine metabolism in MS and EAE (84).

Spermidine shows anti-inflammatory activity *in vitro* by polarizing BV2 microglia toward an M2 phenotype (117) and can bias T-cell differentiation toward Tregs (118). In EAE, spermidine administration reduces disease severity (119), a phenotype later attributed to M2 macrophage polarization and a more anti-inflammatory milieu (120). Another study demonstrated that inhibiting the rate-limiting enzymes ODC1 and SAT1 significantly alleviates EAE, decreases inflammatory cytokine production in draining lymph nodes from antigen-specific cells, and reduces pro-inflammatory immune cell subsets within the CNS (121).

While several studies cite the microbiome as a source of polyamines, direct interrogation of microbially derived polyamines in relation to CNS inflammation is limited. Because T cells can import extracellular polyamines when *de novo* synthesis is impaired (122), gut-derived polyamines could, in principle, shift T-cell polarization toward pro- or anti-inflammatory phenotypes in MS. Supporting clinical relevance, stool levels of the polyamine derivative N1,N12-diacetylspermine were decreased in individuals who transitioned to PMS (5). These decreases in polyamines suggest a loss of potentially protective microbiota or host-derived metabolites in the gut is associated with progression in MS, though causality and tissue sources remain to be defined (5). Collectively, these data indicate that specific polyamines can differentially shape immune responses and may be therapeutically tractable targets.

### Pro- and prebiotics

Probiotic-based strategies have been widely explored for their ability to correct gut dysbiosis and influence MS and EAE outcomes (123–126).

Animal models: In MOG-induced EAE, oral administration of the multi-strain probiotic Vivomixx (including *Lactobacillus*, *Bifidobacterium*, and *Streptococcus thermophilus*) improved motor performance, reduced CNS microgliosis and leukocyte infiltration, and elevated plasma SCFA levels, alongside enrichment of taxa such as Bacteroidota and Actinomycetota (127). Individual strains, including *Clostridium butyricum, Lactobacillus acidipiscis, L. reuteri, Parabacteroides histicola, Akkermansia muciniphila*, and *Anaerobutyricum colihominis*, have also ameliorated EAE by reducing Th1/Th17 responses and associated cytokines (IFN-γ, IL-17) while promoting Treg expansion (28, 123, 126, 128, 129). For example, *L. acidipiscis* increased IL-10, IL-13, and SCFAs in the gut (126), and *Streptococcus thermophilus* enhanced IL-4, IL-5, and IL-10 secretion in MBP-stimulated splenocytes (130). Probiotic treatment often shifts gut composition toward genera negatively associated with MS severity, such as *Bifidobacterium, Prevotella, Lactobacillus, and Sutterella* (128, 131), while *C. butyricum* additionally downregulated p38 MAPK and JNK signaling in the spinal cord (128). In mice, treatment with *Prevotella histicola* suppresses disease by restoring gut permeability and inducing immunoregulatory cells (132). Despite these promising findings, clinical translation remains limited.

Human studies: Probiotic treatment strategy based on the hygiene hypothesis, such as administration of ova from the non-pathogenic helminth, *Trichuris suis*, (TSO) has proven safe and effective in autoimmune inflammatory bowel disease (133, 134). In MS, TSO treatment for three months was safe and reported transient reductions in gadolinium-enhancing lesions in RMS patients, but effects reversed two months after treatment cessation (135). A follow-up study with extended dosing showed only a trend toward MRI improvement and modest immunomodulatory effects on Th1/Th17 balance (133, 134). Although the concept of using a persistent colonic helminth infection to modulate gut immunity and influence MS progression is intriguing, patient preference may limit this approach. Other conventional probiotic trials in MS have yielded mixed results: an 8-species mix [VSL#3, It should be noted that post 2016 VSL#3 probiotic formulation differs from the De Simone Formulation, which was commercially available under the trademark VSL#3^®^ only until 2016 (136)] altered gut microbiota composition and the peripheral immune system during treatment but reverted to baseline within three months of discontinuation (137–139). Short-term interventions in Middle-Eastern cohorts reported modest improvements in depression scores and inflammatory markers, with limited or no effect on EDSS (124, 140). Collectively, probiotics can modulate immune and microbial profiles, but sustained benefits likely require continuous administration and further mechanistic and clinical validation.

Prebiotics are dietary substrates metabolized by gut microbes, with highly diverse functions. They can enhance SCFA production (141), improve mineral absorption (142, 143), and exert systemic effects relevant to MS. Observational data link higher fiber intake to lower disability scores and reduced inflammatory markers in MS cohorts (144, 145). In EAE, fiber-rich diets attenuate disease severity, demyelination, and CNS immune infiltration while elevating gut acetate, propionate, and butyrate (146). Beyond fibers, plant-derived polyphenols exhibit immunomodulatory and neuroprotective properties (147, 148). For instance, polyphenol-rich Jatoba extract reduced EAE severity by limiting macrophage antigen presentation and CD4^+^ T-cell activation, lowering IFN-γ and TNF-α production (149). Similarly, oral pomegranate peel extract (PPE) improved clinical and histological outcomes, suppressed microglial and dendritic cell activation, decreased Th17 differentiation, and promoted Treg induction, alongside microbiome shifts favoring *Prevotellaceae, Lachnospiraceae, and Lactobacillaceae* (150).

## Dietary interventions in multiple sclerosis that can impact the microbiome and its metabolites

Dietary interventions are widely adopted by people living with MS, although evidence-based MS-specific indications are missing. Clinical outcomes remain inconsistent, partly due to small sample sizes (151, 152). EAE studies suggest intermittent fasting alters gut microbiota, a finding partially replicated in a pilot MS trial (153). The Mediterranean diet has shown potential benefits, improving EDSS and fatigue scores after six months (154). The ketogenic diet, characterized by very low carbohydrate and high fat intake, has demonstrated good safety and adherence, and reductions in disability and neurofilament light chain (NfL) levels in MS cohorts (155, 156). However, contrasting evidence indicates higher carbohydrate intake correlates with improvement, while higher fat intake associates with decline in physical performance (157). The modified Paleolithic elimination diet and the low-saturated fat diet, both widely adopted by people living with MS, were directly compared in the randomized WAVES trial (NCT02914964, n=87 RMS). Both diets produced clinically meaningful reductions in fatigue and improvements in physical quality of life, the modified Paleolithic elimination diet additionally improved mental quality of life scores; all outcomes were maintained or further improved at 24 weeks (158). A secondary analysis further demonstrated that both diets significantly reduced weight, BMI, total cholesterol, and LDL, alongside reductions in perceived fatigue, though improvements in fatigue were independent of the metabolic changes, suggesting distinct gut- and immune-mediated mechanisms (158, 159). Both diets share key features, increased vegetable and fiber intake and reduced added sugar, that may favorably modulate gut microbiota composition and SCFA production, though direct microbiome evidence in these cohorts remains limited. Comprehensive analyses, including recent Cochrane reviews, suggests that there is insufficient evidence to determine whether supplementation with antioxidants or other dietary interventions have any impact on MS-related outcomes (152).

Dietary interventions are complex and may influence gastrointestinal physiology and immune function through multiple mechanisms. Adherence remains a challenge, especially with calorie-restricted regimens (151, 152). The specific pathways linking diet, microbiome, and MS pathophysiology are not fully understood. Future research should focus on isolating key dietary components that modulate gut flora and immune responses, paving the way for targeted gastrointestinal-based therapies in MS.

## Discussion

Taken together, current evidence supports a model in which MS-associated microbiota influence CNS autoimmunity through multiple interconnected axes: (i) selective disruption of the mucus and gut-blood barriers, (ii) reprogramming of gut-resident T and B cell responses and innate immune tone, (iii) gut-to-CNS trafficking of microbiota-educated immune cells, including IgA^+^ plasma cells and encephalitogenic T cells, (iv) direct regulation of CNS glia and neuroinflammation by microbial metabolites including SCFAs, bile acids, and polyamines, and (v) peripheral immune signaling that indirectly shapes CNS homeostasis, including a Th17/Treg imbalance. While this framework is compelling, the microbial products and host pathways that drive these effects, and at what level an intervention is most beneficial remain incompletely defined. Additionally the causal role of single bacterial strains vs global microbiota composition in human MS has yet to be firmly established and is difficult to definitively prove it in animal models.

As microbiome-based therapeutics for neurological diseases are emerging, several criteria will be critical for translating preclinical promise into clinical benefit. The MS phenotypic continuum supports interpreting RMS and PMS as clinically dominant expressions of overlapping biology rather than distinct entities. Effective interventions should demonstrate measurable effects on tangible clinical outcomes (symptomatic relapse rate and/or disability worsening) and/or paraclinical outcomes (new lesion formation, lesion enlargement, generalized and local atrophy, microglia activity and white matter integrity) regardless of disease phenotype definitions as RMS or PMS. Mechanistically, these interventions should modulate gut microbiota composition and function, induce durable immunoregulatory effects within the gut mucosa, and exert downstream effects on CNS inflammation, tailored to age- and sex-specific biological differences. Such approaches may not be beneficial for the entire MS population and current evidence can help identifying individuals with the most severe dysbiosis and most likely to benefit from taylored treatment. Approaches such as targeted probiotics, microbial metabolite supplementation, and evidence-based dietary interventions show early promise in meeting these criteria. Realizing their full therapeutic potential will require rigorously designed, adequately powered trials that integrate longitudinal microbiome profiling, immune phenotyping, and validated clinical endpoints, supported by mechanistic biomarkers capable of capturing gut-immune-CNS crosstalk, to establish both efficacy and safety across the full spectrum of MS phenotypes.
